# Interaction of the Vagus Nerve and Serotonin in the Gut–Brain Axis

**DOI:** 10.3390/ijms26031160

**Published:** 2025-01-29

**Authors:** Young Keun Hwang, Jae Sang Oh

**Affiliations:** 1 Department of Medical Sciences, Graduate School, The Catholic University of Korea, Seoul 06591, Republic of Korea; hyg13954@gmail.com; 2 Department of Neurosurgery, Uijeongbu St. Mary’s Hospital, College of Medicine, The Catholic University of Korea, Seoul 06591, Republic of Korea

**Keywords:** gut–brain axis, vagus nerve, serotonin, short-chain fatty acids, nucleus tractus solitaries, dorsal raphe nucleus, locus coeruleus

## Abstract

The gut–brain axis represents an important bidirectional communication network, with the vagus nerve acting as a central conduit for peripheral signals from the various gut organs to the central nervous system. Among the molecular mediators involved, serotonin (5-HT), synthesized predominantly by enterochromaffin cells in the gut, plays a pivotal role. Gut-derived serotonin activates vagal afferent fibers, transmitting signals to the nucleus tractus solitarius (NTS) and modulating serotonergic neurons in the dorsal raphe nucleus (DRN) as well as the norepinephrinergic neurons in the locus coeruleus (LC). This interaction influences emotional regulation, stress responses, and immune modulation. Emerging evidence also highlights the role of microbial metabolites, particularly short-chain fatty acids (SCFAs), in enhancing serotonin synthesis and vagal activity, thereby shaping gut–brain communication. This review synthesizes the current knowledge on serotonin signaling, vagal nerve pathways, and central autonomic regulation, with an emphasis on their implications for neuropsychiatric and gastrointestinal disorders. By elucidating these pathways, novel therapeutic strategies targeting the gut–brain axis may be developed to improve mental and physical health outcomes.

## 1. Introduction

The gut–brain axis (GBA) is a complex, bidirectional communication network that integrates signals between the various gut organs tract and the central nervous system (CNS). It encompasses multiple pathways, including neural, hormonal [1], and immune mechanisms [2], which collectively maintain homeostasis and influence both physical and mental health. At the heart of this intricate network lies the vagus nerve, the primary conduit for transmitting peripheral signals from the gut to the brain and vice versa. This pathway is essential for autonomic regulation, gut motility, and immune responses, playing a pivotal role in mediating the effects of gut-derived molecules on the CNS [3].

Serotonin (5-hydroxytryptamine, 5-HT) is one of the most significant neurotransmitters involved in gut–brain communication. Approximately 90% of the body’s serotonin is synthesized in the gut, predominantly by enterochromaffin (EC) cells. This gut-derived serotonin not only regulates local intestinal functions, such as peristalsis and secretion, but also exerts systemic effects by activating vagal afferent fibers [4]. These fibers relay serotonergic signals to the NTS in the brainstem, where they are processed and transmitted to higher brain regions [5], including the DRN and LC. Through these connections, serotonin influences emotional regulation, stress responses, and immune modulation [6].

Recent research has highlighted the role of the gut microbiota in modulating serotonin production [7,8]. Gut bacteria produce metabolites such as short-chain fatty acids (SCFAs), which have been shown to enhance the expression of tryptophan hydroxylase 1 (TPH1), the rate-limiting enzyme in serotonin synthesis [9]. SCFAs also modulate vagal activity and serotonin transporter (SERT) expression, further strengthening the microbiota–gut–brain axis [10]. This interaction underscores the significance of gut-derived signals in influencing brain function and systemic immunity.

Understanding the mechanisms underlying serotonin-mediated communication via the vagus nerve is crucial for elucidating its role in health and disease. The vagus nerve is a key part of the brain–gut axis, and recent studies have suggested that antidepressants may contribute to the relief of depressive symptoms by modulating vagus nerve activity. Furthermore, the dysregulation of this axis has been implicated in a range of conditions, from irritable bowel syndrome (IBS) and inflammatory bowel disease (IBD) to neuropsychiatric disorders such as anxiety and depression [11,12]. Collectively, we aim to integrate insights from neurophysiology and microbiology to explore the critical role of serotonin, the vagus nerve, and the gut–brain axis in maintaining physiological balance and their potential as therapeutic targets to improve mental and physical health.

## 2. Vagus Nerve

The vagus nerve is the tenth cranial nerve, the longest cranial nerve, and a key component of the parasympathetic nervous system. It originates in the medulla oblongata of the brainstem and extends to major organs, including the heart, lungs, spleen, and gastrointestinal tract, where it communicates in two directions to regulate a variety of physiological processes, including heart rate, blood pressure, digestion, and inflammation. The vagus nerve plays an important role in maintaining the homeostasis of the autonomic nervous system and is also closely associated with neuropsychiatric disorders such as depression and epilepsy. Vagus nerve stimulation (VNS) is an important adjunctive treatment for patients with treatment-resistant depression (TRD) and has been shown to significantly reduce depressive symptoms and improve remission rates when used in combination with conventional antidepressants [13]. Mechanistically, VNS may contribute to its antidepressant properties by increasing monoamine transmission and exerting anti-inflammatory effects [14]. However, VNS requires a surgical procedure and is expensive, so antipsychotic medications are also used as an adjunct; however, due to side effects, probiotics have recently been used. One study found that a combination of probiotics (*Lactobacillus acidophilus*, *Bifidobacterium bifidum*, and *Streptococcus thermophilus*) significantly reduced the frequency of major depressive episodes in participants after 8 weeks, suggesting that probiotics are effective in treating major depressive disorder [15].

The vagus nerve is composed of unmyelinated sensory afferent fibers (approximately 80–90% of all nerve fibers) and some myelinated efferent fibers (approximately 10–20%) [16,17,18,19], and afferent fibers play an important role in transmitting sensory information from various visceral organs to the brain. These afferent signals originate from tension receptors in the stomach and esophagus, as well as chemoreceptors and mechanoreceptors in the gastric mucosa [20], and are transmitted to the nucleus tractus solitarius (NTS) of the medulla oblongata via the tuberous ganglion of the vagus nerve. The NTS is the center for processing visceral sensory and chemical signals and projects signals to the hypothalamus, locus coeruleus (LC), amygdala, and cerebral cortex, contributing to the regulation of the autonomic nervous system and emotional and cognitive responses. Afferent fibers are divided into intra-intestinal and extra-intestinal fibers, with intra-intestinal fibers responding to the mechanical movement, distension, and changes in chemical composition at the receptors in the walls of the gastrointestinal tract, as well as the extra-intestinal fibers responding to the mechanical movement, distension, and changes in chemical composition at the receptors in the pancreas, liver, portal vein, and lungs. Regarding the metabolic role of vagal afferent innervation [21,22,23], the afferent fibers in the pancreas regulate glucose concentration and insulin secretion [24], and the chemoreceptors in the liver and portal vein sense fatty acid, amino acid, and glucose concentrations to maintain metabolic homeostasis. The portal vein is the main pathway for transporting serotonin produced in the gut to the liver, and some serotonin signals to the NTS via vagal afferent fibers. In addition, the LC is involved in the release of norepinephrine [25] and interacts with the serotonergic system to regulate emotional responses. Afferent fibers originating from the lungs, heart, and kidneys connect and coordinate physiological functions such as respiration, heart rate, and blood pressure with the central nervous system, and vagal afferent fibers play a key role in the regulation of the gut–brain axis and the autonomic nervous system.

The efferent fibers of the vagus nerve originate from the Nucleus Ambiguus (NAm) in the medulla oblongata and the Dorsal Motor Nucleus of the Vagus (DMV). The NAm primarily regulates motor signals and sends signals to the muscles of the palate, pharynx, and larynx to perform functions such as swallowing and vocalization. It also sends parasympathetic signals that reduce heart rate via the vagal cardiac branches. The DMV, on the other hand, is the main parasympathetic efferent signaling pathway of the vagus nerve, which gives rise to innervating fibers that are distributed to several visceral organs, including the stomach, bronchi, small intestine, and pancreas [26]. These nerves play an important role in the control of intestinal muscle contraction and secretion, as well as in the regulation of intestinal motility.

Afferent and efferent nerves interact closely, and the vagus nerve maintains the harmonious functioning of the autonomic nervous system through bi-directional regulation between the brain and internal organs. For example, sensory signals from the stomach and intestines are transmitted via afferent fibers and processed by the brainstem and limbic system, which in turn transmit parasympathetic signals to the organs via efferent fibers to control bowel motility, digestive secretions, and heart rate. This bidirectional regulation of the vagus nerve is not limited to the heart, lungs, and digestive system but is also involved in immune response, inflammation control, and emotional and cognitive functions, helping to maintain homeostasis and regulate a variety of physiological and neuropsychiatric conditions.

## 3. Synthesis and Secretion of Serotonin in the Gut

### 3.1. 5-HT Synthesis in Intestinal Chromaffin-Affinity Cells (EC Cells)

**Figure 1:**
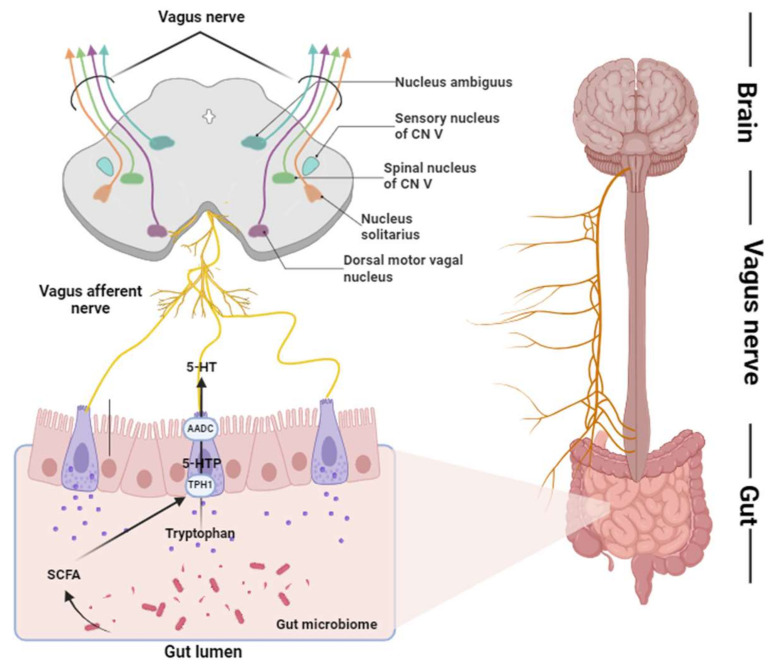
Serotonin-mediated communication within the gut–brain axis via the vagus nerve. This figure illustrates the bidirectional interactions between the gut and brain, emphasizing the role of serotonin (5-HT) and the vagus nerve. In the gut, tryptophan, derived from dietary sources, is converted into serotonin through the action of tryptophan hydroxylase (TPH1) and aromatic L-amino acid decarboxylase (AADC). The gut microbiome influences serotonin production by releasing short-chain fatty acids (SCFAs), which modulate TPH1 expression and serotonin synthesis. Serotonin produced in the gut activates vagal afferent fibers, which transmit signals to the nucleus tractus solitarius (NTS) in the brainstem. The NTS integrates serotonergic signals and projects to higher-order brain regions involved in autonomic regulation, mood, and immune function. Key brainstem nuclei, including the dorsal motor vagal nucleus, participate in efferent signaling back to the gut, completing the feedback loop. This dynamic system highlights the essential role of serotonin in gut-brain communication and the influence of gut microbiota in regulating neural and physiological responses.

Enterochromaffin (EC) cells are specialized epithelial cells located in the mucosal layer of the gastrointestinal tract and found in high densities primarily in the small intestine (especially the jejunum and ileum) and large intestine. These cells are also present in small amounts in the duodenum and stomach and are intimately connected to the enteric nervous system (ENS), responding to mechanical, chemical, and microbial stimuli from the intestinal environment. One of the primary functions of EC cells is to synthesize and secrete serotonin (5-hydroxytryptophan, 5-HT) [27], which plays a key role in gut motility, secretion, immune response, and neural regulation via the brain–gut axis (Figure 1). The synthesis of serotonin proceeds using an essential amino acid called tryptophan as a precursor. First, the EC cells absorb food-derived tryptophan from the blood and transport it into the cells [28]. Absorbed tryptophan is converted to 5-hydroxytryptophan (5-HTP) by tryptophan hydroxylase (TPH), and in the EC cells, the TPH1 isoenzyme, which is expressed in peripheral tissues, is responsible for this process. In the intestine, TPH1-dependent 5-HT biosynthesis occurs in the enterochromaffin cells of the mucosal epithelium and in the mast cells of mice and rats, accounting for 90% of intestinal 5-HT production [29,30]. TPH2 is located in the neurons of the ENS and CNS and accounts for the remaining 10% of 5-HT production in the gut [31,32] (Figure 1). 5-HTP is then converted to serotonin by aromatic L-amino acid decarboxylase (AADC) [29,33]. Serotonin has a very short half-life in the brain [34]. Active serotonin is transported into the synaptic space, and inactive serorotonin is metabolized in and out of the cell. Serotonin also plays an important role in regulating macrophage polarization, specifically affecting the balance between M1 and M2 phenotypes. The 5-HT2B and 5-HT7 receptors mediate the anti-inflammatory effects of serotonin, with the 5-HT2B receptor preferentially expressed on M2 macrophages [35]. Serotonin increases the expression of M2-associated genes, such as SERPINB2 and THBS1, while simultaneously downregulating the expression of M1-associated genes by inhibiting the release of pro-inflammatory cytokines [36]. These changes are essential for maintaining tissue homeostasis and resolving inflammation [35]. However, the dysregulation of serotonin signaling in macrophages can lead to pathological conditions such as chronic inflammatory diseases, autoimmune diseases, and cancer. Therefore, it is important to maintain a balance between M1 and M2 macrophage polarization for an effective immune response [37]. Serotonin synthesized by EC cells acts on the enteric nervous system and smooth muscle cells to regulate intestinal peristalsis and is released in response to a variety of stimuli. It also contributes to the regulation of immune responses and systemic signaling via the vagus nerve, which plays an important role in the function of the gastrointestinal tract and the central nervous system as a whole.

### 3.2. Serotonin Synthesis in the Gut Microbiome

SCFAs produced in the intestinal lumen are transported into colonic epithelial cells either in their undissociated form or via monocarboxylate transporters (MCTs). Specifically, the monocarboxylate transporter 1 (MCT1), a pH-dependent hydrogen-coupled transporter, and the sodium-coupled monocarboxylate transporter 1 (SMCT1) facilitate the uptake of SCFAs in their dissociated form [38]. SCFAs (mainly butyrate) in the gut lumen stimulate Tph1 expression in enterochromaffin cells, which then leads to increased 5-HT production by the enterochromaffin cells [39]. Microorganisms such as *Lactobacillus* and *Bifidobacterium* modulate tryptophan metabolism to promote serotonin production and increase tryptophan availability in the gut [40,41]. SCFAs can affect serotonin by modulating the expression of SERTs and serotonin receptors (5-HT1A, 5-HT2B, and 5-HT7) [42]. SCFAs affect brain function through direct interactions with G protein-coupled receptors (GPCRs) such as FFAR2 and FFAR3 [43]. These GPCRs are found in both the central and peripheral nervous systems, with particularly high densities in the peripheral organs [44,45]. SCFAs also communicate with the brain via the afferent vagus nerve to activate neurons in areas of the central nervous system [46]. SCFAs also affect the serotonergic system, and one of their main mechanisms is by modulating tryptophan synthesis. Tryptophan is the sole precursor of serotonin biosynthesis, and its circulating levels depend on dietary intake and tryptophan metabolism by gut bacteria [47]. However, most of the free tryptophan in the blood is consumed via the kynurenine pathway, and the remaining tryptophan must cross the BBB for serotonin synthesis to occur in the central nervous system [48]. Systemic levels of tryptophan are closely linked to inflammatory states, and inflammatory cytokines can induce the expression of indoleamine 2,3-dioxygenase and tryptophan-2,3-dioxygenase, which are involved in KYN synthesis in tryptophan metabolism, limiting the availability of tryptophan for serotonin synthesis [49,50]. On the other hand, SCFAs may indirectly increase tryptophan availability through their anti-inflammatory effects in the systemic circulation. SCFAs maintain cytokine balance by decreasing inflammatory cytokines (TNF-α, IL-1β, IL-6) and increasing levels of anti-inflammatory and regulatory cytokines such as IL-10. In doing so, SCFAs have the potential to increase the availability of tryptophan, which is required for serotonin biosynthesis [51,52].

## 4. Serotonin Produced in the Gut and Delivered to the Afferent Fibers of the Vagus Nerve

The afferent fibers of the vagus nerve originate from terminals located in various layers of the intestinal wall, including the mucosal proprioceptive plate, the muscularis propria, and the fascial plexus, with nerve cell bodies located in the tuberous ganglia. These fibers project via the vagus nerve to the solitary ganglion, where they play an important role in processing sensory information and relaying it to the central autonomic network (CAN) [53]. The terminals of afferent fibers are divided into intravascular and mucosal endings based on their morphological structure and functional role. Intravascular endings are located between the iliac plexus, longitudinal muscle layer, and circular muscle layer and are specialized for sensing muscle tension in the gastrointestinal tract. Mucosal endings, on the other hand, are located near the tips of villi or the luminal opening of the intestine and respond to chemical and mechanical stimuli, including nutrients and irritants [54].

**Figure 2:**
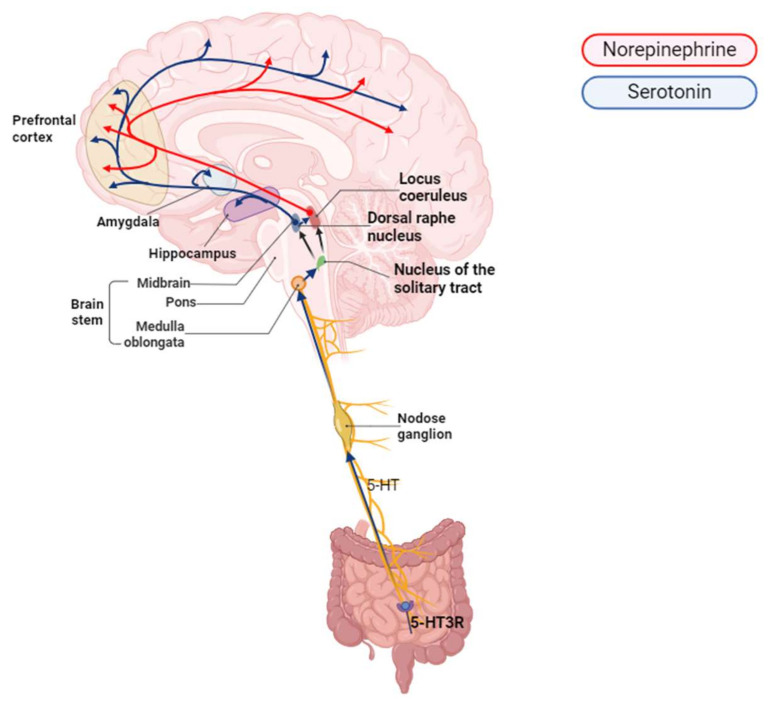
Serotonin and norepinephrine pathways connecting the gut and brain through the vagus nerve. This diagram highlights the dual involvement of serotonin (blue pathways) and norepinephrine (red pathways) in gut–brain communication via the vagus nerve. Serotonin produced in the gut interacts with 5-HT3 receptors located on vagal afferent fibers, transmitting signals to the nucleus of the solitary tract in the brainstem. The nucleus tractus solitarius (NTS) integrates these serotonergic signals and projects them to higher brain regions, including the dorsal raphe nucleus, locus coeruleus, hippocampus, and cortex, where they influence mood, cognition, and stress responses. In parallel, norepinephrine pathways originating from the LC modulate various cortical and subcortical regions, playing a key role in arousal, attention, and immune regulation. The interplay between these neurotransmitter systems is essential for maintaining homeostasis, as serotonin influences norepinephrine release and vice versa, creating a feedback loop that supports adaptive responses to internal and external stimuli.

In particular, the EC cells in the intestine are the major source of serotonin production in the gastrointestinal mucosa, releasing 5-HT in response to gut microbes and mechanical and chemical stimuli [55,56] (Figure 2). The released 5-HT activates 5-HT3 receptors (5-HT3R) on the afferent fibers of the vagus nerve [57,58] and is relayed through synapses or neuronal cell bodies at the nodose ganglion. The nodose ganglion is the first major aggregation point for vagal afferent signals, processing neural information about intestinal stimuli [59], which are processed in the NTS and then spread to the amygdala, LC, and other areas of the brain [60,61] (Figure 2). It also affects cardiac vagal outflow by modulating reflexes through various receptor subtypes, including 5-HT1A, 5-HT2, and 5-HT3, particularly within the NTS [62]. The terminals of afferent fibers serve a variety of functions, including chemoreceptors, mechanoreceptors, and osmotic receptors, in which gut hormones such as ghrelin, cholecystokinin (CCK), and glucagon-like peptide-1 (GLP-1) influence nerve activity via chemoreceptors [63,64]. The gut microbe *Bacteroides thetaiotaomicron* induces 5-HT production through the activation of the EC cells, which in turn mediates the function of the enteric axis by transmitting signals from the submucosal plexus to interact with the enteric nervous system [65].

## 5. Signal Processing and Transmission from the NTS to the DRN and Its Connection to the Brain

The rapid processing and transmission of gut signals relies on the structure and activity of the vagus afferent system, and the branching pattern of the vagus afferent nerves is essential for the performance of selective biological functions. These afferent neurons have a pseudo-bipolar structure and relay signals to the NTS in the brainstem. The NTS is a major integration and distribution center for afferent signals and plays a critical role in a variety of physiological processes, including autonomic nervous system regulation and HPA axis responses [66,67]. The NTS functions as a key component of the central autonomic network, which relays signals to various brain regions, including LC and the paraventricular hypothalamus. The NTS uses serotonin and glutamate released by afferent fibers as its primary neurotransmitters [68,69] and processes signals specifically through the postsynaptic AMPA and NMDA receptors [70]. These processed signals are then relayed in a highly organized form, with some going to the DRN and others to the LC and hypothalamus, each performing specific functions [63,71,72]. Signals sent to the DRN have an important influence on serotonin release. The DRN is composed of a population of serotonergic neurons and is connected to the limbic system (particularly the amygdala and hippocampus) and prefrontal cortex, where it is involved in higher-order neural activity, including emotional regulation and stress response [73,74,75,76]. NTS signaling activated by gut stimuli promotes serotonin release in the DRN, which contributes to stress relief and immune regulation [77,78].

## 6. The Role of the NTS in Signaling to the DRN and LC and in Regulating the Release of Serotonin and NE

The NTS is strongly connected to both the DRN and LC and plays a key role in regulating serotonin and NE release in these regions, respectively. The DRN contributes to mood and stress regulation through serotonin release, and signaling from the NTS modulates immune and autonomic nervous system responses by regulating the serotonin release associated with gut stimuli [77,78]. In patients with irritable bowel syndrome (IBS), increased serotonin release from EC cells triggers the over-activation of mesenteric sensory neurons, leading to increased mucosal sensitivity, and these signals can further exacerbate emotional regulation and stress responses via the DRN and LC [79].

The LC is a major center for NE release, which regulates attention and arousal, while also influencing autonomic nervous system responses. Signals from the NTS either induce or inhibit sympathetic activation in the LC, which in turn influences immune responses via the peripheral immune organs such as the spleen [80,81]. In addition, the NTS can be modulated by external stimuli such as probiotics. A specific probiotic (Saccharomyces boulardii) has been shown to normalize signaling in the NTS and gut–brain axis, contributing to enhanced function of the mucosal barrier and modulating intestinal signaling [82]. Furthermore, in addition to the DRN and LC, the NTS is connected to higher-order brain regions such as the amygdala, hippocampus, and prefrontal cortex, making it a key hub for gut stimulation to influence cognition and emotional function [83]. As such, the NTS regulates the release of serotonin and NE, as well as coordinating the integrated responses of the autonomic and immune systems, which together form a critical neurological network that influences emotional and stress responses.

By further characterizing the interaction of the DRN and LC, we can discuss how serotonin and NE work in concert to achieve autonomic nervous system regulation and stress response. Interactions between both the serotonin and NE systems play an important role in the pathophysiology and treatment of anxiety disorders. These interactions are critical to understanding the neurobiological mechanisms underlying these disorders and the efficacy of pharmacologic interventions.

## 7. Serotonin and Norepinephrine in Disease

### 7.1. The Role of Serotonin in MDD and ADS

A lack of serotonin in the brain can exacerbate the negative emotions associated with major depressive disorder (MDD), including depressed mood, self-blame, criticism, disgust, fear, anxiety, hostility, irritability, and loneliness [84]. Serum serotonin concentrations in MDD patients are significantly lower than in healthy controls, resulting in a serotonin deficiency in MDD patients [85,86] and reduced serotonin transporter (SERT) availability [85,86]. A meta-analysis of in vivo neuroimaging and postmortem studies found that SERT binding was significantly reduced in the striatum, amygdala, and brainstem but not in the thalamus or hippocampus [87,88]. The severity of this depression has been shown to be associated with older age and more severe depression, with greater reductions in SERT, particularly in the amygdala and striatum [88]. Decreased serotonergic neurotransmission may contribute to affective disorders such as MDD and AD, as well as other neurotransmitter systems [89].

### 7.2. The Role of NE in MDD and ADS

Research on major depressive disorder (MDD) has highlighted the important role that NE and its receptors play in the pathophysiology of the disorder. It has been shown that NE transmission is altered in MDD patients and that increased NE transporter (NET) availability in the thalamus correlates with attention deficits [90]. Conversely, reduced NET levels were observed in the LC in depressed subjects [91]. The involvement of α-adrenergic receptors in MDD has been suggested, but direct evidence is limited [92]. Increased binding of agonist ligands has been observed at the α2-adrenergic autoreceptors on the NE neuron cell bodies, indicating higher function of these NE autoreceptors and thus lower noradrenergic neurotransmission in MDD [93]. The α2-adrenergic autoreceptors occur presynaptically on noradrenergic and serotonergic nerve terminals and exert an inhibitory effect on neurotransmitter release upon stimulation. Recent research has focused on β-adrenergic receptors, particularly the β-3 subtype, which may promote stress resilience and offer new therapeutic approaches [94].

### 7.3. Interaction of Serotonin and Norepinephrine

Chronic antidepressant treatment decreases the sensitivity of norepinephrine-sensitive adenylate cyclase, leading to the downregulation of beta-adrenergic receptors. This process requires intact serotonergic input, indicating a critical interaction between the serotonin and NE systems [95,96,97]. Serotonin can regulate the number and function of beta-adrenergic receptors, and lesions of the serotonergic nervous system increase the number of beta-adrenergic receptors and alter receptor function [96]. Serotonin and NE neurons have mutual inhibitory and excitatory effects on each other. Serotonin can inhibit the firing of NE neurons, and NE can inhibit serotonin neurons [98,99]. This interaction is important for the behavioral effects of antidepressants because it affects the overall neurotransmitter balance and response to treatment [99]. Serotonin–norepinephrine reuptake inhibitors (SNRIs) and selective serotonin reuptake inhibitors (SSRIs) can enhance antidepressant effects by affecting both the serotonin and NE systems. This dual action is particularly useful in treatment-resistant depression [95,100,101]. For example, combining an SSRI with an agonist that enhances NE release may improve treatment outcomes in resistant cases [100,101]. As such, the reciprocal regulation of both the serotonin and NE systems influences a variety of behavioral and clinical outcomes, including mood regulation, anxiety, and overall mental health. Effective antidepressant treatment is critical to balancing these interactions for optimal outcomes.

## 8. Interspecies Differences in 5-HT Receptor Subtype Expression and Limitations of Analytical Methods

Interspecies differences in 5-HT receptor subtype expression are important for understanding the variations reported in the literature but are not yet fully understood (Table 1). For example, 5-HT2B receptor mRNA is absent in the rat brain but is widely distributed in the human brain, suggesting that 5-HT2B receptors may perform unique functions in the human central nervous system [102]. Furthermore, in primates, 5-HT1B receptors are absent, with 5-HT1D receptors serving a similar function, whereas in rodents, 5-HT1B receptors are distributed in the basal ganglia, amygdala, and hypothalamus and influence behavior [103]. In the nucleus in humans, 5-HT4 receptors have lower binding levels compared to rats, showing that receptor distribution is species-specific [104], and 5-HT1E receptors are identified in humans but not in rodents, further emphasizing interspecies differences. In addition, the similarity between the 5-HT6 and 5-HT2 receptors is also species-specific, indicating diversity in receptor homology [105].

A variety of methods are used to investigate 5-HT receptor subtype expression, but these methods have several limitations. For example, direct sequencing and pyrosequencing of individual cDNA clones can introduce significant sampling error due to limited clone numbers, which can obscure or overestimate expression differences [106]. Ligand binding technologies and molecular biological methods are confounded by the complexity of receptor pharmacology or struggle to accurately categorize multiple receptor subtypes [107]. Limitations of TaqMan reverse transcriptase–polymerase chain reaction and selective radioligand binding methods have been reported, with low receptor expression levels in mice and different distributional characteristics in humans complicating direct comparisons between species [108].

**Table 1:** The Role of Serotonin Receptors in the Brain.

Receptors	Expression Sites	Receptor Function	References
5-HT1A	Brainstem, prefrontal cortex, neocortex, hippocampus	Regulates the brain’s mood, anxiety, cognition, sleep, and pain perception.	[109,110,111]
5-HT1B	Cerebral cortex, hippocampus, substantia nigra, brainstem raphe nuclei	Regulates overall serotonin release in the brain by inhibiting serotonin release upon activation. Reduces serotonin release in various brain regions, including the dorsal raphe and raphe median nucleus.	[112,113]
5-HT1F	Neocortex	Activated by a tryptan compound approved for the treatment of migraine.	[114]
5-HT2A	Cortex	Regulates vascular tone, contributes to mood, cognition, and psychedelic effects. Psychedelic substances known for their potent psychoactive effects primarily interact with 5-HTR2A.	[115,116]
5-HT2C	Cortex	Unlike 5-HT2A receptors, 5-HT2C receptors are preferentially distributed in subcortical areas including basal ganglia and related structures	[117]
5-HT3	Larynx	Forms the only 5-HT receptor subtype that functions as a ligand-gated ion channel (i.e., an ionotropic receptor).	[117]
5-HT4	Hippocampus	Controls brain physiological functions such as learning and memory, feeding and mood behavior, as well as gastrointestinal transit, couples several G proteins, notably Gs, G13, and in somei cell lines, the q signaling pathway.	[118]
5-HT6	Prefrontal cortex and insula	Regulates choline function in the brain, modulating GABAergic neurotransmission.	[119]

## 9. Discussion

Beyond its well-established functions in the gut, serotonin serves as a critical messenger in signaling pathways mediated by the vagus nerve. It facilitates the transmission of peripheral sensory information to the central nervous system, where it influences key brain regions such as the NTS, DRN, and LC. This intricate network highlights the dual role of serotonin as a bridge between gut-derived signals and brain-directed responses.

The regulation of serotonin within the gut–brain axis is profoundly influenced by gut microbiota. Metabolites like SCFAs not only enhance serotonin synthesis by upregulating TPH1 but also modulate vagal activity and SERT expression. This interplay demonstrates how microbial communities act as a dynamic regulatory layer in serotonin signaling. Additionally, vagal afferent fibers, with their serotonin receptor subtypes, refine this signaling cascade by translating local GI signals into centralized neural outputs that modulate mood, stress responses, and immune function.

Disruptions in serotonin regulation within this axis can lead to significant physiological and pathological outcomes, ranging from altered gut motility and immune dysregulation to neuropsychiatric disorders such as anxiety and depression. Understanding the nuances of serotonin’s role in these contexts offers a valuable opportunity for therapeutic innovation. Interventions targeting gut serotonin synthesis, microbial modulation, and vagus nerve signaling could provide a multi-faceted approach to restoring balance in the gut–brain axis.

In conclusion, serotonin’s regulation within the gut–brain axis underscores its essential role in maintaining physiological harmony and its potential as a therapeutic target. Future research should focus on elucidating the precise molecular mechanisms linking serotonin to vagal and microbial pathways, aiming to develop novel treatments for conditions arising from gut–brain dysregulation. The centrality of serotonin in this network offers a promising avenue for enhancing our understanding of the gut–brain axis and translating these insights into impactful clinical applications.

## 10. Current Challenges and Future Prospects

The intricate complexity of the gut–brain axis presents significant challenges in elucidating the precise mechanisms underlying serotonin-mediated communication. A major hurdle lies in understanding the bidirectional interactions among gut-derived serotonin, vagal nerve pathways, and central nervous system targets such as the NTS, DRN, and LC. Additionally, the interspecies differences in serotonin receptor expression and the limitations in analytical methods hinder the translation of preclinical findings to clinical applications. Current therapeutic strategies targeting this axis remain in their infancy, with limited tools to modulate serotonin pathways effectively without unintended systemic effects.

Looking ahead, advancements in multi-omics technologies, high-resolution imaging, and neuroengineering are poised to unravel the complex interplays among the gut microbiota, serotonin synthesis, and vagal signaling. Personalized medicine approaches, integrating patient-specific microbiota profiles and serotonin receptor phenotypes, hold promise for tailored interventions. Furthermore, the development of microbiota-based therapies, such as targeted probiotics and postbiotics, could revolutionize the treatment of neuro-psychiatric and gastrointestinal disorders. By addressing these challenges, future research can pave the way for innovative therapeutic strategies that harness the gut–brain axis to improve mental and physical health outcomes.

## Abbreviations

GBA	Gut–brain axis
SCFAs	Short-chain fatty acids
NTS	Nucleus tractus solitariu
DRN	Dorsal raphe nucleus
LC	Locus coeruleus
NE	Norepinephrine
